# PCV3-associated disease in the United States swine herd

**DOI:** 10.1080/22221751.2019.1613176

**Published:** 2019-05-16

**Authors:** Bailey Arruda, Pablo Piñeyro, Rachel Derscheid, Ben Hause, Emily Byers, Kate Dion, Duane Long, Chris Sievers, Jon Tangen, Todd Williams, Kent Schwartz

**Affiliations:** a Department of Veterinary Diagnostic and Production Animal Medicine, Iowa State University, Ames, IA, USA; b Cambridge Technologies, Worthington, MN, USA; c Prestage Farms, Inc, Clinton, NC, USA; d The Hanor Company of Wisconsin, LLC, Enid, OK, USA; e Swine Health Care, Mexico, IN, USA; f Swine Vet Center, St Peter, MN, USA; g Pipestone Veterinary Service, Ottumwa, IA, USA

**Keywords:** Porcine circovirus, reproductive failure, porcine dermatitis and nephropathy syndrome, myocarditis, encephalitis

## Abstract

Porcine circovirus-associated disease encompasses multiple disease syndromes including porcine circovirus 2 systemic diseases, reproductive failure, and porcine dermatitis and nephropathy syndrome. Until recently, porcine circovirus 2 was the only species associated with the porcine circovirus-associated disease. In this report, diagnostic investigations of thirty-six field cases submitted from multiple production systems, numerous sites and varied geographic locations demonstrated porcine circovirus 3 within lesions by *in situ* hybridization including fetuses with myocarditis, weak-born neonatal piglets with encephalitis and myocarditis, from cases of porcine dermatitis and nephropathy syndrome, and in weaned pigs with systemic periarteritis. Porcine circovirus 3 was detected by PCR in numerous fetuses and perinatal piglets at high viral loads (trillions of genome copies per mL of tissue homogenate). Samples from all cases in this study were assayed and found negative for porcine circovirus 2 by PCR. Metagenomic sequencing was performed on a subset of reproductive cases, consisting of sixteen fetuses/fetal sample pools. PCV3 was identified in all pools and the only virus identified in fourteen pools. Based on these data, porcine circovirus 3 is considered a putative cause of reproductive failure, encephalitis and myocarditis in perinatal piglets, porcine dermatitis and nephropathy syndrome, and periarteritis in swine in the United States.

## Introduction

Porcine circovirus (PCV) is a group of circular, single-stranded DNA viruses belonging to the family *Circoviridae*, genus *Circovirus* [1]. Circoviruses are the smallest known autonomously replicating viruses [1]. The genome encodes two major open reading frames (ORFs) where ORF1 encodes a replication-associated protein (*rep*) and ORF2 encodes the viral capsid (*cap*) protein that determines the antigenic characteristics of the virus [1].

PCV is common in the United States and global swine herds, and until recently, the only two species identified were PCV1 and PCV2 [2]. PCV1 was first identified as a cell culture contaminate and has not been associated with disease [3]. In contrast, PCV2 is an economically important pathogen that has been associated with a broad range of clinical diseases including porcine circovirus 2 systemic disease (PCV2-SD), respiratory and enteric disease, reproductive failure, and porcine dermatitis and nephropathy syndrome (PDNS) [4–7].

Pigs with clinical signs and lesions of PCV2-SD were first reported in Canada, and this syndrome was subsequently associated with PCV2 in the mid-to-late 1990s [5,8]. Shortly thereafter, epidemics of severe systemic disease characterized by rapid loss of condition, high mortality, and failure to thrive were documented in Europe, Asia, and the United States [9–14]. Histologic lesions consistently included lymphoid depletion and lymphohistiocytic or granulomatous inflammation in multiple organ systems in which PCV2 was readily detected by immunohistochemistry. Subsequently, PCV2 infection was associated with other syndromes collectively termed porcine circovirus-associated disease (PCVAD), which includes PCV2-SD, interstitial pneumonia, PDNS, and reproductive failure [6,7]. The broad range of clinical diseases of PCVAD may be influenced by concurrent infection with other swine pathogens or risk factors, as infection with PCV2 alone is commonly subclinical [7].

Reproduction of PCV2-SD under controlled conditions has usually included coinfection with pathogens such as porcine parvovirus (PPV), porcine reproductive and respiratory syndrome virus (PRRSV), or immune stimulation [15]. PDNS is thought to be the result of an immune-complex deposition and is included as a PCVAD because of the association between PDNS lesions and PCV2 detection. However, there are a subset of cases in which lesions in the kidney and/or skin are consistent with PDNS without detection of PCV2. Gross lesions of PDNS are characterized by prominent well-demarcated red to dark purple macules and papules most prevalent on the hindquarters and dependent portions of the pig [7]. Renal lesions include cortical pallor and petechiae. Histologic lesions of PDNS in kidney are characterized by vasculitis and fibrinous glomerulitis and interstitial nephritis with tubular degeneration and necrosis [6,7]. Skin lesions are characterized by epidermal necrosis, dermal hemorrhage, and leukocytoclastic vasculitis [16].

Naturally occurring PCV2-associated reproductive failure has mainly been reported in newly established production facilities with first parity gilts [17–19]. Histologic lesions in fetuses can include lymphocytic or lymphohistiocytic myocarditis, with the highest viral titers and the highest proportion of infected cells in the heart [2]. Criteria for the diagnosis of PCV2-associated reproductive disease have included later-term abortions and stillbirths, extensive fibrosing and/or necrotizing myocarditis, and the presence of high amounts of PCV2 in myocardial lesions and other fetal tissues [20]. However, these classic criteria are insufficient to accurately diagnosis a majority of PCV2-associated reproductive failure cases [21]. Quantitative PCR has been shown to be a more sensitive diagnostic method and is now used routinely to diagnose PCV2-associated reproductive failure cases in the absence of fetal cardiac lesions [21].

In 2015 and 2016, a new PCV species was detected in sows with lesions consistent with PDNS and concurrently in mummified fetuses on a sow farm [22], as well as in pigs with lesions of cardiac and multisystemic inflammation in the United States [23]. Metagenomic sequencing and analysis, showed this new virus was genetically distinct from PCV2, with only 48% amino acid identity in the *rep* protein and 26% amino acid identity in the *cap* protein between the two viruses [22,23]. Thus, this new PCV species was designated PCV3 [22]. The virus has since been identified in multiple countries, including Germany [24], Japan [25], Korea [26], Russia [27], China [28], Thailand [29], Italy [30], Spain [31], Denmark [32], South Korea [33], Poland [34], Brazil [35], and Sweden [36]. While testing is somewhat limited to date, the finding of PCV3 in retrospective samples indicates that this species of circovirus has likely been circulating in swine populations decades prior to the initial detection and case reports [36].

Phylogenetic analysis of contemporary and retrospective samples has shown a consistent mutation in certain amino acids of the *cap* protein. Accordingly, it has been proposed that mutations in amino acids 24 and 27 of the *cap* protein could be potential molecular markers to classify PCV3 into three clades: PCV3a, PCV3b and PCV3c [37]. PCV3a has been further divide into three subclades (PCV3a1, PCV3a2 and PCV3a3) based on the evolutionary relationship and other molecular features of the *cap* protein [37].

Following the initials reports [22,23], PCV3 has been detected in herds reporting clinical signs of reproductive failure [27,30,35,36], PDNS [27], diarrhea [38], and respiratory disease [29,38,39]; however, these reports do not provide strong evidence that PCV3 is associated with clinical disease because they report limited information on tests and testing of other concomitant swine pathogens, lack pathologic evaluation, and/or do not detect PCV3 in lesions through *in situ*. Detection of an endemic agent does not suffice for disease diagnosis. The array of clinical syndromes occurring in different production systems from which there is concurrent detection of PCV3 by PCR and *in situ* in lesions has not been reported, leaving many to question the clinical relevance of an apparently endemic PCV3 in the United States and global swine herds. Accordingly, this report investigates the role of PCV3 and its cell tropisms in various clinical syndromes in samples submitted for diagnostic investigation from different productions systems through the aggregation of clinical history, clinical signs, and diagnostic results that include histopathology and *in situ* hybridization (ISH) assays.

## Materials and methods

### Selection of cases

All cases were submitted to the Iowa State University Veterinary Diagnostic Laboratory (ISU-VDL) for routine diagnostic investigation. The ISU-VDL is a National Animal Health Laboratory Network accredited laboratory that receives more than 80,000 cases per year, 75% of which are the porcine origin. Approximately 10,000 porcine cases include gross and histologic evaluation of tissues. Reproductive cases were selected based on the detection of PCV3 by qPCR at cycle quantification (Cq) values at or below 30.0 in the absence of other known viral causes of reproductive failure in swine. Perinatal infection cases were selected based on the detection of PCV3 by qPCR and ISH in lesions present in weak-born piglets. Weaned pig case selection was based on the presence of consistent histologic lesions that included periarteritis and arteritis and detection of PCV3 by qPCR and ISH. All weaned pig cases were reviewed by BA as the assigned diagnostic pathologist.

### Gross and histologic evaluation

Gross evaluation of all fetuses, intact pigs, and fresh and fixed tissue was performed by a diagnostic pathologist. Findings were recorded in the case record. Tissues were fixed in 10% neutral buffered formalin, processed by standard technique and stained by hematoxylin and eosin (H&E) technique. Histologic evaluation was performed by a diagnostic pathologist. Sections from these same paraffin blocks were also used for RNAscope® ISH.

### PCV3 *in situ* hybridization

RNA ISH was performed using the RNAscope® 2.5 HD Reagent Kit -RED (catalog no. 322350 Advanced Cell Diagnostics, Newark, CA) and the RNAscope® 2.5 HD Reagent Kit – BROWN (catalog no. 322300 Advanced Cell Diagnostics, Newark, CA) according to the manufacturer's instructions for formalin-fixed paraffin-embedded samples (document numbers: 322452, 322360 and 322310 Advanced Cell Diagnostics, Newark, CA). Paraffin blocks stored at room temperature (RT) from each selected case were retrieved and 4 µm sections were trimmed and mounted on Superfrost® Plus slides (catalog no. 48311–703 VWR, Radnor, PA). Slides were then dried overnight at RT. Next slides were heated for 1 h at 60°C and afterwards deparaffinized with two consecutive 5 min immersions in xylene and two consecutive 1 min immersions in 100% alcohol. Slides were air dried, treated with RNAscope® Hydrogen Peroxide at RT for 10 min and then rinsed with distilled water. Slides were then immersed in the prepared RNAscope® 1X Target Retrieval Reagent for 15 min at 100°C. This was followed with a distilled water rinse, 100% alcohol immersion for three minutes, air drying of the slides and application of a hydrophobic barrier using the ImmEdge™ pen around each tissue section. Slides were then treated with RNAscope® Protease Plus for 30 min at 40°C in the HybEZ™ Oven followed by a distilled water rinse. Preheated probes were then dispensed on to the samples which then hybridized for 2 h at 40°C in the HybEZ™ Oven. The RNAscope® probe targeting PCV3 RNA (catalog no. 463961 or 530431) along with the RNAscope® positive control probe Ss-UBC (catalog no. 400641) and RNAscope® negative control probe DapB (catalog no. 310043) used were designed and synthesized by Advanced Cell Diagnostics. After probe hybridization, six rounds of amplification were performed (RNAscope ® 2.5 AMP 1–6) followed by incubation with either red or brown chromogenic detection solution for 10 min at RT. Slides were counterstained with a 50% hematoxylin solution for 2 min, rinsed and dipped three times in 0.02% ammonia water followed by a tap water rinse Slides with the red detection were then dried at 60°C for 15 min and cooled down at RT for 5 min. Finally, slides were quickly submerged into xylene and immediately coverslipped after applying two drops of EcoMount (catalog no. EM897L Biocare Medical, Pacheco, CA). Slides with brown detection were dehydrated through one change of 70% alcohol and two changes of 95% alcohol for 2 min each before placing in xylene for 5 min. Slides were finally coverslipped with a mounting medium suitable for use with xylene. Slides were visualized by a diagnostic pathologist using an Olympus BX43 bright-field microscope (Olympus Corporation, Tokyo, Japan).

### PCV3 and PCV2 qPCR

All samples were tested at the ISU-VDL for the presence of PCV3 and PCV2. Tissues were minced using a sterile scissors and forceps, transferred to a 50 mL conical tube with Earle’s Balanced Salt Solution (Sigma-Aldrich, St. Louis, MO) to make a 10% weight/volume homogenate, the conical tube was then placed in a Geno/Grinder® (SPEX® SamplePrep, Metuchen, NJ) at 1000 RPM for 2 min followed by centrifugation at 4200 × *g* for 10 min at 4°C and the resulting supernatant was transferred into a 5 mL snap-cap tube. DNA extraction from the tissue homogenate was performed using the MagMAX-96 Pathogen RNA/DNA kit (Applied Biosystem™, MA, USA) with KingFisher™ Flex 96 Deep-Well Magnetic Particle Processor (Thermo Fisher Scientific, Waltham, MA) following the manufacturer’s instructions. DNA extracts were used to detect the conserved region of the capsid gene of PCV3 using TaqMan™ Fast Virus 1-step Master Mix (Life Technologies, MA, USA) with specific primers previously described [17]. The ORF1 of PCV2 was detected using forward primer 5′-TGGCCCGCAGTATTCTGATT-3′ and reverse primer 5′-CAGCTGGGACAGCAGTTGAG-3′ with the probe 5′-56-FAM-CCAGCAATC-ZEN-AGACCCCGTTGGAATG-3IABkFQ-3′ [40]. Internal control RNA (Xeno™, Life Technologies Corporation) was included in the master mix to monitor PCR amplification and detection of PCR inhibition. Two positive extraction controls, one negative extraction control, and a negative amplification control were included. RT-qPCR was performed (7500 Fast Real-Time PCR System, Applied Biosystems®, Foster City, CA) with the following cycling conditions: one cycle at 50°C for 5 min, one cycle at 95°C for 20 min, 40 cycles at 95°C for 3 s, and 60°C for 30 s. Samples with Cq values <37 for either species were considered positive. The PCV3 genome copy number per mL of tissue homogenate was calculated from standard curve created by the ten-fold serial dilution of plasmid DNA containing full genome of PCV3 isolate 29160 (GenBank accession no. KT869077). The equation for PCV3 genome copy number per mL of tissue homogenate was 10^((Cq−53.795)/−3.6391)^.

### PRRSV, PPV, and APPV qPCR

All testing was performed at the ISU-VDL. Tissue processing and nucleic acid extraction were performed as above. For PRRSV, RT-qPCR was performed on nucleic acid extracts using a commercially available NA and EU PRRSV-specific PCR assay (Applied Biosystems™ TaqMan® NA and EU PRRSV Reagents, Thermo Fisher Scientific). The assay contained multiple primers and probes to detect North American (Type 2) and European (Type 1) strains. For PPV qPCR was performed as above with forward primer 5′-CCAAAAATGCAAACCCCAATA-3′ and reverse primer 5′-TCTGGCGGTGTTGGAGTTAAG-3′ with the probe 5′-56-FAM-CTTGGAGCCGTGGAGCGAGCC-IA Black FQ-3′ [41]. For APPV, RT-qPCR was performed as above with forward primer 5′-TGCCTGGTATTCGTGGC-3′ and reverse primer 5′-TCATCCCATGTTCCAGAGT-3′ with the probe 5′-5-FAM-CCTCCGTCTCCGCGGCTTCTTTGG-3BHQ_2-3′ [42]. Internal controls, equipment and cycling conditions are as described above. Samples with Cq values <37 for either PRRSV genotype were considered positive. Samples with Cq values <35 for PPV and APPV were considered positive.

### Metagenomic sequencing

In reproductive failure Case nos. 1, 3, and 7, metagenomic sequencing was performed as previously described [40,41]. First-strand cDNA synthesis of viral DNA was performed with the Superscript III first-strand synthesis kit in accordance with the manufacturer’s instructions with previously published primers [42]. Amplification of the double-stranded cDNA was performed with primers identical to those used for first-strand synthesis but lacking the random hexamer and TaKaRa DNA polymerase. Reads were assembled *de novo* and contigs were identified by BLASTX. Template assembly was performed using PCV3-IT/MN2017 (accession no. MF162299) as a reference. For most assemblies, a gap was present in the intergenic region downstream of the *cap* and *rep* ORFs. To complete the genome sequence, this region was amplified by PCR using primers PCV3 733 Forw (5′-AAACGCGGGAAGCTTGTGC) and PCV3 1782 Rev (5′-ACCCGCCTAAACGAATGG). These primers were designed based on the assembled PCV3 genome sequence adjacent to the gap in the assembly in order to amplify the region of the genome encompassing the gap. The resulting amplicon was sequenced using an Illumina MiniSeq with libraries prepared using the Nextera XT library preparation kit. Amplicon sequencing reads were combined with reads generated by metagenomic sequencing of the original sample to generate the complete genome sequence.

In reproductive failure Case nos. 19, 20, 21, and 22, metagenomics sequencing was performed using Illumina technologies and NextEra XT library preparation. Each sample was processed in two methods to enrich for either RNA or DNA viruses. Sequences were aligned to *Sus scrofa* to remove host and remaining sequences were assembled *de novo* and analyzed by BLASTN and BLASTX.

### PCV3 open reading frame 2 sequencing and phylogenic analysis

All samples were tested at the ISU-VDL. DNA extraction from tissues was performed using the MagMAX-96 Pathogen RNA/DNA kit (Applied Biosystem™, MA, USA) with KingFisher™ Flex 96 Deep-Well Magnetic Particle Processor (Thermo Fisher Scientific, Waltham, MA) following the manufacturer’s instructions. QScript XLT Custom One-Step RT–PCR kit was used with Quanta Custom Tough Mix and two primer sets developed at the ISU-VDL. The first primer set consisted of forward primer 1 5′-GTGTACAATTATTGCGTTGGG-3′ and reverse primer 1 5′-AAAACACAGCCGTTACTTCACC-3′. The second primer set consisted of forward primer 2 5′-GCTTTGTCCTGGGTGAGCG-3′ and reverse primer 2 5′-CCTGCGGCATCAAAACACG-3′. Sequencing was performed (ABI 2720 thermal cycler, Applied Biosystems®, Foster City, CA) with the following cycling conditions: one cycle at 94°C for 3 min, 45 cycles at 94°C for 30 s, 55°C for 30 s, and 68°C for 45 s, one cycle at 68°C for 7 min, and 1 cycle at 4°C. Successful performance of the assay was determined when a PCR product of 444 and 643 bp was obtained from primer set one and two, respectively.

For phylogenetic analysis, a full PCV3 ORF2 sequence from 11 clinical cases was aligned with PCV3 reference strain sequences obtained from GenBank (https://www.ncbi.nlm.nih.gov/nucleotide/). PCV3 clade reference strains were selected from US strains with the exception of PCV3b as a US strain was not available (PCV3a: PCV3USA/MO/2015 (KX778720), PCV3/USA/29160/NC/2016 (NC031753), PCV3/USA/MN20162016 (KX898030); PCV3b: PCV3/CN/Jiangxi-S1/2017 (MF589133); and PCV3c: PCV3/USA/SD2016/2016 (KX966193)). Multiple sequence alignment and sequence comparisons were carried out using the ClustalW algorithm by Geneious R9 software (Biomatters Ltd, Auckland, NZ). Phylogenetic analysis was completed using PAUP (phylogenetic analysis using parsimony) version 4.0b10 by the maximum parsimony (MP) method. The MP tree was executed using a heuristic search with stepwise option. The clade of trees was estimated with 1000 replicates of bootstrap (BT) analysis. The trees were visualized with Geneious R9 software based on full sequence of the ORF2. All sequences were translated and PCV3 clustering was evaluated based on the amino acid composition of the ORF2 as previously proposed [37].

## Results

### Reproductive failure cases

#### Cases and clinical history

A summary of reproductive failure cases is presented in Table 1. Between January and November 2018, PCV3 was detected by qPCR in fetal thoracic tissues (pooled heart and lung) in 22 cases that included 36 fetuses or fetal groups. Cases usually included multiple fetuses and often fetuses from multiple litters or sow groups. These 22 cases originated from 20 different sites located in Iowa, Indiana, Nebraska, Kansas, South Dakota, Michigan, Minnesota, North Carolina, or Ohio. Information from submission forms often reported an increased mummy and stillbirth rate, most commonly in low parity sows. Two cases specifically reported a 5% mummy rate. Rarely weak-born pigs or decreased farrowing rate were also reported. The single batch farrow farm in this study (Case no. 1), reported multiple batches affected with most of the losses occurring over two farrowings. Initially, gilt litters were reported to be affected with more multiparous sows having affected litters in the next batch. It was reported that up to 35 litters of 100 total litters per farrowing were affected, with around three-fourths of the pigs stillborn. In Case nos. 19–21, gilts were sourced from the same multiplier. These three geographically distinct and unrelated commercial farms reported widespread reproductive failure in gilts that included early embryonic death, increased mummy rate, and decreased farrowing rate (77–83% compared to historical 88–91%).

**Table 1.** Summary of diagnostic assay results for reproductive failure cases.

			^ ^	PCR Cq	^ ^	^ ^	^ ^	^ ^
Case no.	Site state	Site ID	PCV3 Cq^a^ – group^b^	PCV2^c^	PPRSV^d^	PPV^e^	NGS^f^	ISH^g^
1	IN	A	20.6	U^h^	U	U	PCV3	Heart and kidney
2	MN	B	19.7 – A	U	NP^i^	U	NP	NP
			14.1 – B					
			U – C, D					
3	IA	C	9.4	U	U	U	PCV3	Heart not kidney
4	NE	D	15.2 – A	U	NP	NP	NP	NP
			U – B					
			12.2 – C					
			28.3 – D					
			24.8 – E					
			30.0 – F					
5	OH	E	17.8	U	NP	U	NP	NP
6	IA	C	14.8 – A	U	U	U	PCV3	Heart, kidney, placenta – A
			30.7 – B					
			10.8 – C					Heart and kidney – C
			U – D, E, F					
7	NE	F	18.0	U	U	U	PCV3	Heart and kidney
8	KS	G	24.7 – A	U	NP	U	NP	NP
			U – B, C					
9	MN	H	13.6 – A	U	U	U	NP	Heart not kidney
			30.0 – B					
			33.3 – C					
			14.5 – D					
10	MN	B	13.1	U	U	U	NP	NP
11	NE	I	20.6	U	NP	NP	NP	NP
12	IA	J	17.9	U	U	U	NP	NP
13	IN	K	21.7 – A	U	U	U	NP	NP
			27.8 – B					
14	IA	L	13.8	U	U	NP	NP	NP
15	NC	M	19.8	U	U	U	NP	NP
16	NE	N	24.6	U	U	U	NP	NP
17	SD	O	10.0	U	U	U	NP	NP
18	IA	P	U – A, B	U	U	U	NP	NP
		* *	17.5 – C					
		* *	13.5 – D					
19^j^	MI	Q	23.5	U	U	U	PCV3	NP
20	MI	R	14.0	U	U	U	PCV3	NP
21	MI	S	20.9	U	U	U	PCV3	NP
22	MN	T	11.4 – A	U	U	U	PCV3	NP
			14.0 – B					
			26.9 – C					

^a^PCV3 Cq: Porcine circovirus 3 cycle quantification value.

^b^Group: Individual fetuses or pooled fetal group identification.

^c^Porcine circovirus 2.

^d^Porcine reproductive and respiratory syndrome virus.

^e^Porcine parvovirus.

^f^Metagenomics sequencing detection of PCV3.

^g^ISH: *In situ* hybridization; tissues in which PCV3 was detected by RNAscope® ISH assay.

^h^U: Undetected after cycle cut-off.

^i^NP: Not performed.

^j^Case No. 19–21: The same gilt source was used by three different commercial farms.

#### Pathology and PCV3 ISH

**Figure 1.**
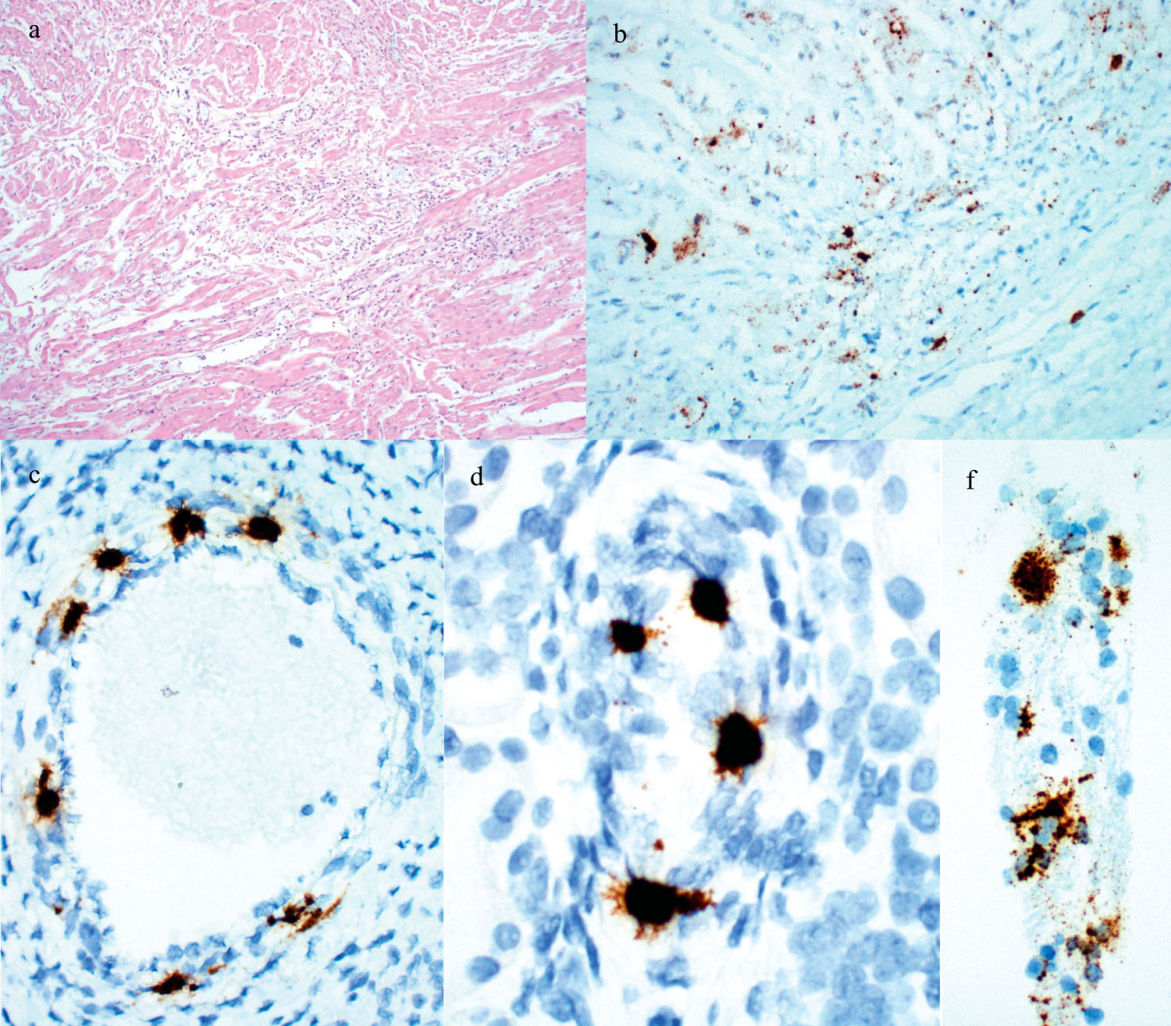
Histologic lesions and detection of PCV3 by ISH in reproductive failure cases. (a) Fetal heart with lymphocytic myocarditis from Case no. 6 fetal group C. (b) PCV3 replicating in fetal heart from Case no. 6 fetal group C. (c) PCV3 replicating in the smooth muscle of an arteriole in the heart from a fetus in Case no. 7. (d) PCV3 replicating in the smooth muscle of an arteriole in the kidney from a fetus in Case no. 7. (e) PCV3 replicating in trophoblasts of the placenta from Case no. 6 fetal group A.

Gross examination of stillborn fetuses was diagnostically unremarkable. Fetuses which had died earlier *in utero*, often with *in utero* dehydration or mummification without abortion, had a crown-to-rump length that varied from 7 to 17 cm. Histologic examination of the heart, lung, spleen, liver, kidney and placenta was unremarkable in a majority of cases. Lymphocytic myocarditis was observed in fetuses of two cases that originated from the same site (Figure 1(a)).

PCV3 was detected by ISH in 5 out of 5 cases assayed. The most abundant labeling was present in a section of heart that also had inflammation (Figure 1(b)). Replication of PCV3 was most commonly noted in the smooth muscle of arteries in the heart and kidney and within cardiac myocytes (Figure 1(c,d)). In one case placenta was tested and found positive for the presence of PCV3 in trophoblasts (Figure 1(e)).

#### PCV3 detection in tissues by qPCR

PCV3 was detected in multiple fetuses and usually 50% or more of fetuses within litters or pooled fetal groups. The Cq value of fetal thoracic tissue ranged between 9.4 (1.58 × 10^12 ^gc/mL) to 33.3 (4.28 × 10^5 ^gc/mL). Three cases had litters or groups of pooled fetal thoracic tissue with Cq values at or above 30.0 (3.46 × 10^6 ^gc/mL). In these cases, multiple fetal groups were submitted and tested individually with the lowest Cq values found to be 12.2 (2.69 × 10^11 ^gc/mL), 10.8 (6.53 × 10^11 ^gc/mL), and 13.6 (1.11 × 10^11^ gc/mL). The mean and median Cq value of all positive fetal thoracic tissues tested (*n* = 36) was 19.26 (3.09 × 10^9 ^gc/mL) and 17.95 (7.08 × 10^9 ^gc/mL), respectively. The standard deviation of Cq values was 6.87. In one case multiple sample types across three positive litters were tested including fetal thoracic tissue, fetal thoracic fluid, liver and kidney. In this case, fetal thoracic fluid had the lowest Cq value of any sample type (Supplementary Table 1). Concurrent testing of kidney and fetal thoracic tissue occurred in three cases. Kidney had a slightly higher Cq value than fetal thoracic tissue (Supplementary Table 1).

#### Viral pathogen detection in tissues by qPCR

PCV2 was not detected by qPCR in any fetus or fetal group. PRRSV was not detected by RT-qPCR in any case tested (*n* = 17). PPV was not detected by qPCR in any case tested (*n* = 19).

#### Metagenomic sequencing

A summary of metagenomic sequencing results is presented in Supplementary Table 2. The MiSeq runs generated a total of 6,858,986 reads. Reads were assembled *de novo*. PCV3 was identified in sixteen fetal pools assayed from eight cases. The complete PCV3 genome was recovered from at least one sample in each case with 99–100% genome similarity to a reference strain, when analyzed by BLASTX. In fourteen samples, the remaining reads showed no similarity to any known eukaryotic virus. An adventitious agent along with PCV3 was identified in two samples: torque teno sus virus (TTSuV-1) and porcine cytomegalovirus (PCMV). Assemblies from RNA processing methods revealed no evidence of RNA viral infection (data not shown).

### Perinatal infection cases

#### Cases and clinical history

Between March and August of 2018, PCV3 was detected in three cases that included suckling piglets. Two cases were submitted a month apart from the same high-health, newly populated “start-up” herd in Missouri reporting poor estrus activity, periods of higher abortion rates, and increase in fetal mummies. The first of these was fresh and fixed cerebrum from a 12-day-old pig reported with tremors. Submitted in the second case was fresh and fixed tissues including cerebrum (fresh only), heart, thymus, lymph node, spleen, tonsil, lung, kidney, liver, small intestine, and large intestine from an 8-day-old piglet taken from a litter born with multiple mummified fetuses. The third case originated from a genetic multiplier herd in North Carolina and consisted of fresh and fixed tissues including cerebrum, lung, heart, and kidney from three 1-day-old piglets from a gilt litter, with a third of the pigs displaying tremors. Other pigs in the litter were reported to be weak and not nursing well.

#### Pathology and PCV3 ISH

**Figure 2.**
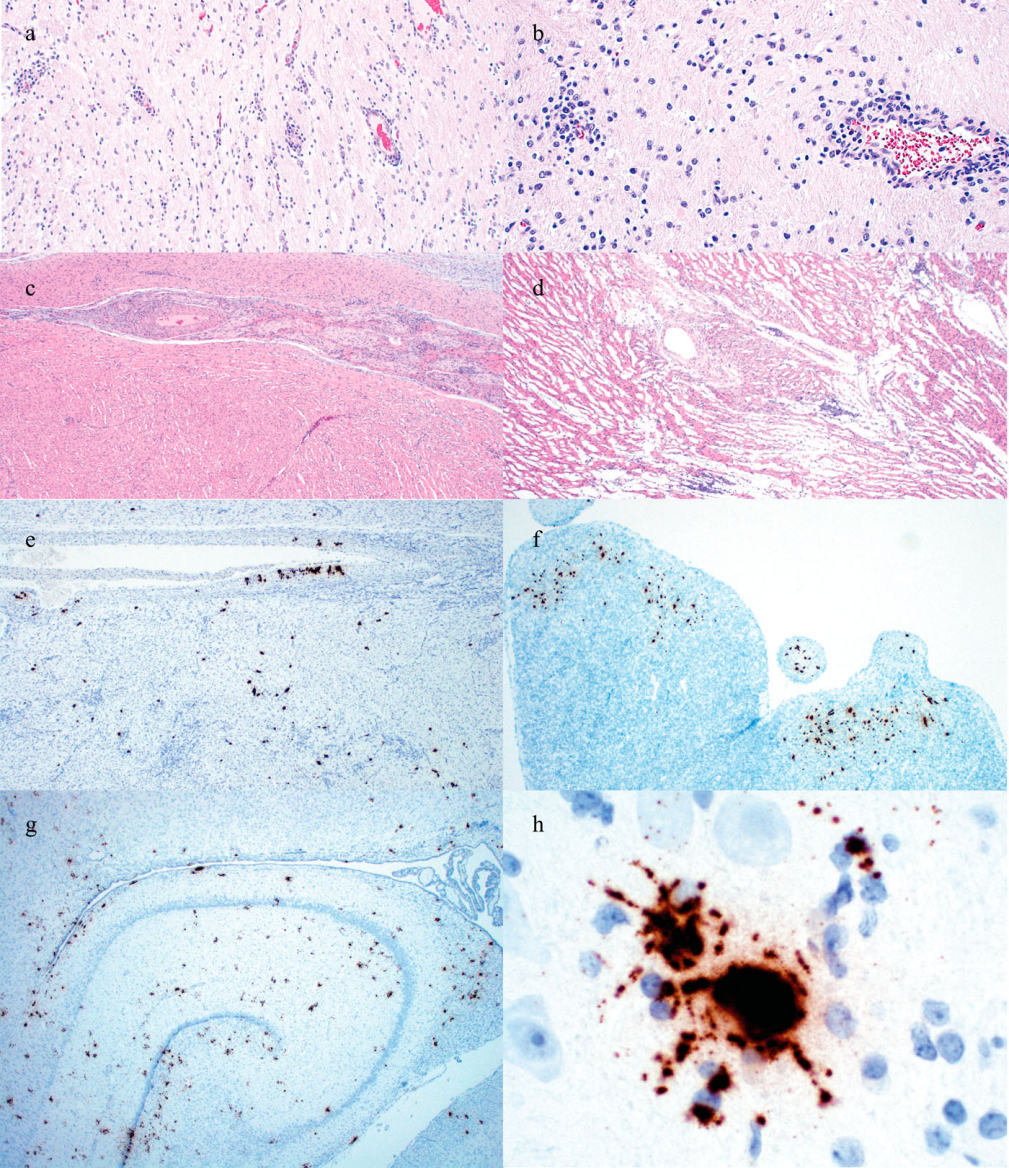
Histologic lesions and detection of PCV3 by ISH in perinatal infection cases. (a) Lymphocytic perivascular cuffing in the cerebrum of the 12-day-old piglet from Case no. 1. (b) Lymphocytic perivascular cuffing and gliosis in the cerebrum of a 1-day-old piglet from Case no. 3. (c) Lymphocytic myocarditis and perivasculitis in the 8-day-old piglet from Case no. 2. (d) Lymphocytic myocarditis in a piglet from Case no. 3. (e) PCV3 replicating in cardiac myocytes and the smooth muscle of an artery of a piglet from Case no. 2. (f) PCV3 replicating in cardiac myocytes of a piglet from Case no. 3. (g) PCV3 replicating in neurons and ependymal cells in the cerebrum at the level of the hippocampus of a piglet from Case no. 3. (h) PCV3 in the body and axons of a neuron in the cerebrum of the piglet from Case no. 1.

Gross examination of fresh and fixed tissues was diagnostically unremarkable. Gliosis and lymphocytic perivascular cuffing were noted in the cerebrum in the first and third case (Figure 2(a,b)). Lymphocytic perivasculitis and myocarditis were present in the second and third case (Figure 2(c,d)).

PCV3 was detected by ISH in all three cases. Replication of PCV3 was noted in smooth muscle of arteries in the heart (Figure 2(e)), cardiac myocytes (Figure 2(e,f)), the white and grey matter of the cerebrum, smooth muscle of arterioles in the cerebrum, ependymal cells, numerous neurons (Figure 2(g,h)), and smooth muscle of arterioles of the kidney and renal tubular epithelium.

#### PCV3 detection in tissues by qPCR

PCV3 was detected by PCR in all three cases at very low Cq values. PCV3 was detected in the brain from piglets of all three cases at Cq values of 12.2 (2.69 × 10^11 ^gc/mL), 13.3 (1.34 × 10^11 ^gc/mL), and 7.5 (5.27 × 10^12 ^gc/mL), respectively. PCV3 was also detected in pooled lung and spleen (Cq 13.7; 1.04 × 10^11 ^gc/mL) and liver (Cq 19.3; 3.01 × 10^9^ gc/mL) from the piglet in the second case. PCV3 was detected in pooled lung and heart (Cq 14.4; 6.69 × 10^10 ^gc/mL) from the piglets in the third case.

#### Viral pathogen detection in tissues by qPCR

PCV2 was not detected by qPCR in any case. PRRSV was not detected by RT-qPCR in the single case assayed. APPV was detected by RT-qPCR in the brain of piglets from the first (Cq 28.1) and third case (Cq 27.8) that reported tremors.

#### Metagenomic sequencing

Metagenomic sequencing of the cerebrum from Case no. 2, identified PCV3 alone. The MiSeq runs generated a total of 2,790,668 reads. Reads were assembled *de novo*, resulting in 296 contigs. One contig consisted of 1617-bp composed of 1615 reads that mapped to the reference, PCV3-IT/MN2017 (accession no. MF162299) via templated assembly with 98–99% genome similarity, when analyzed by BLASTX. The remaining reads mapped to host (*Sus scrofa*) genome.

### Weaned pig cases

#### Cases and clinical history

A summary of weaned pig cases is presented in Table 2. Between February and October of 2018, PCV3 was detected in eleven cases from nine different sites. Five of the cases came from three sites that were supplied by the same sow farm. In all cases, pigs ranged in age from 3- to 10-weeks-old but were most commonly 3 to 6 weeks of age. Clinical signs varied and included acute deaths, poor condition and dermatopathy in 0.25% of pigs from a “start-up” herd.

**Table 2.** Summary of diagnostic assay results for weaned pig cases.

Case no.	Site state	Site ID	Age (wk)	Clinical signs	Gross	Histopathology	PCR Cq^a^			ISH^e^
							PCV3^b^	PCV2^c^	PRRS^d^	
1	IA	K	3	Dermatopathy	Palpable purpura	Haired skin: Vasculitis and perivasculitis, fibrinoid and leukocytoclastic with epidermal necrosis	23.4	U^f^	U	Skin: Adipocytes in panniculus LN^g^: Rare lymphocytes
2^h^	IA	L	3	Derma-topathy	Palpable purpura	Haired skin: Vasculitis and perivasculitis, fibrinoid and leukocytoclastic with epidermal necrosis Heart, kidney, and mesenteric arteries: Periarteritis and arteritis, lymphohistiocytic and plasmacytic	26.5	U	NP^i^	SI^j^: Adipocytes of mesentery, mesothelial cells, rare crypt cells, rare enterocytes, rare smooth muscle of tunica muscularis, smooth muscle of arteries LI^k^: Smooth muscle of arteries Skin: Endothelial cells, epithelium of hair follicle, adipocytes in panniculus, epithelium
3	IA	M	5	Sudden death	DU^l^	Heart, kidney, mesentery of spleen: Periarteritis and arteritis, lymphohistiocytic and plasmacytic	25.0	U	NP	Lung: Low numbers of mesothelial cells of pleura, pneumocytes, smooth muscle of arteries Liver: Not detected Heart: Occasional cardiac myocytes, smooth muscle of arteries, lymphocytes surrounding arteries Kidney: Smooth muscle of artery Spleen: Mesothelial cells of mesentery, smooth muscle of arteries in spleen and mesentery, adipocytes in mesentery, smooth muscle of trabeculae, lymphocytes
4	IA	M	8	Resp^c^	DU	Heart: Lymphohistiocytic myocarditis, multifocal, mild with segmental periarteritis Kidney: Interstitial nephritis, lymphohistiocytic, multifocal, moderate	25.8	U	31.4	Liver: Hepatocytes in low numbers commonly in clusters Heart: Rare cardiac myocytes
5	IA	M	9	Resp	DU	Kidney: Periarteritis, lymphohistiocytic and plasmacytic	27.0	U	U	Spleen: Lymphocytes Lung: Not detected Kidney: Rare renal tubular epithelium; adipocytes surrounding kidney
6	IA	N	10	Sudden death	DU	Heart: Periarteritis, lymphohistiocytic	28.0	U	23.3	Heart: Smooth muscle of artery
7	IA	O	3	Enteric	Fluid filled LI	Heart: Periarteritis and arteritis, lymphohistiocytic and plasmacytic Large intestine; mesocolon: Periarteritis and arteritis, lymphohistiocytic and plasmacytic	22.7	U	U	Heart: Cardiac myocytes and smooth muscle of arteries LI: Scattered smooth muscle cells of the tunica muscularis; lymphocytes of GALT Mesocolon: Smooth muscle of arteries LN: Low numbers of lymphocytes Thymus: Not detected Liver: Rare hepatocytes
8	MN	P	6	Resp	Fibrinous epicarditis	Heart, kidney, and mesocolon: Perivasculitis, lymphocytic Heart: Myocarditis, lymphohistiocytic and fibrinous epicarditis	23.2	U	U	Heart: Smooth muscle of arteries; cardiac myocytes LI: Smooth muscle of tunica muscularis; rare colonocytes, adipocytes; smooth muscle of arteries in mesocolon Liver: Rare hepatocytes; smooth muscle of artery
9	NC	Q	5	Resp	DU	Heart and kidney: Periarteritis and arteritis, lymphohistiocytic	23.6	U	U	Kidney: Smooth muscle cells of artery and mononuclear inflammatory cells Heart: Rare cardiac myocytes
10	IL	R	NA	Poor condition	DU	Myocarditis and periarteritis	19.9	U	U	Heart: Smooth muscle cells of an artery; rare cardiac myocytes
11	IL	S	4	Resp	DU	A: Myocarditis, lymphocytic Spleen; mesentery: Arteritis, focal B: Myocarditis, periarteritis, arteritis Kidney: Periarteritis and arteritis	27.2-A 22.1-B	U	31.1-B	A: Rare cardiac myocytes and renal tubular cells B: Cardiac myocytes, adipose surrounding kidney, and smooth muscle of arteries in the heart and kidney

^a^Cq: Cycle quantification value.

^b^Porcine circovirus 3.

^c^Porcine circovirus 2.

^d^Porcine reproductive and respiratory syndrome virus.

^e^ISH: *In situ* hybridization; tissues in which PCV3 was detected by RNAscope® ISH assay.

^f^U: Undetected after cycle cut-off.

^g^LN: Lymph node.

^h^Case No. 2-5: Pigs originated from the same sow source as site ID K.

^i^NP: Not performed.

^j^SI: Small intestine.

^k^LI: Large intestine.

^l^DU: Diagnostically unremarkable.

^m^Resp: Respiratory.

#### Pathology and PCV3 ISH detection

**Figure 3.**
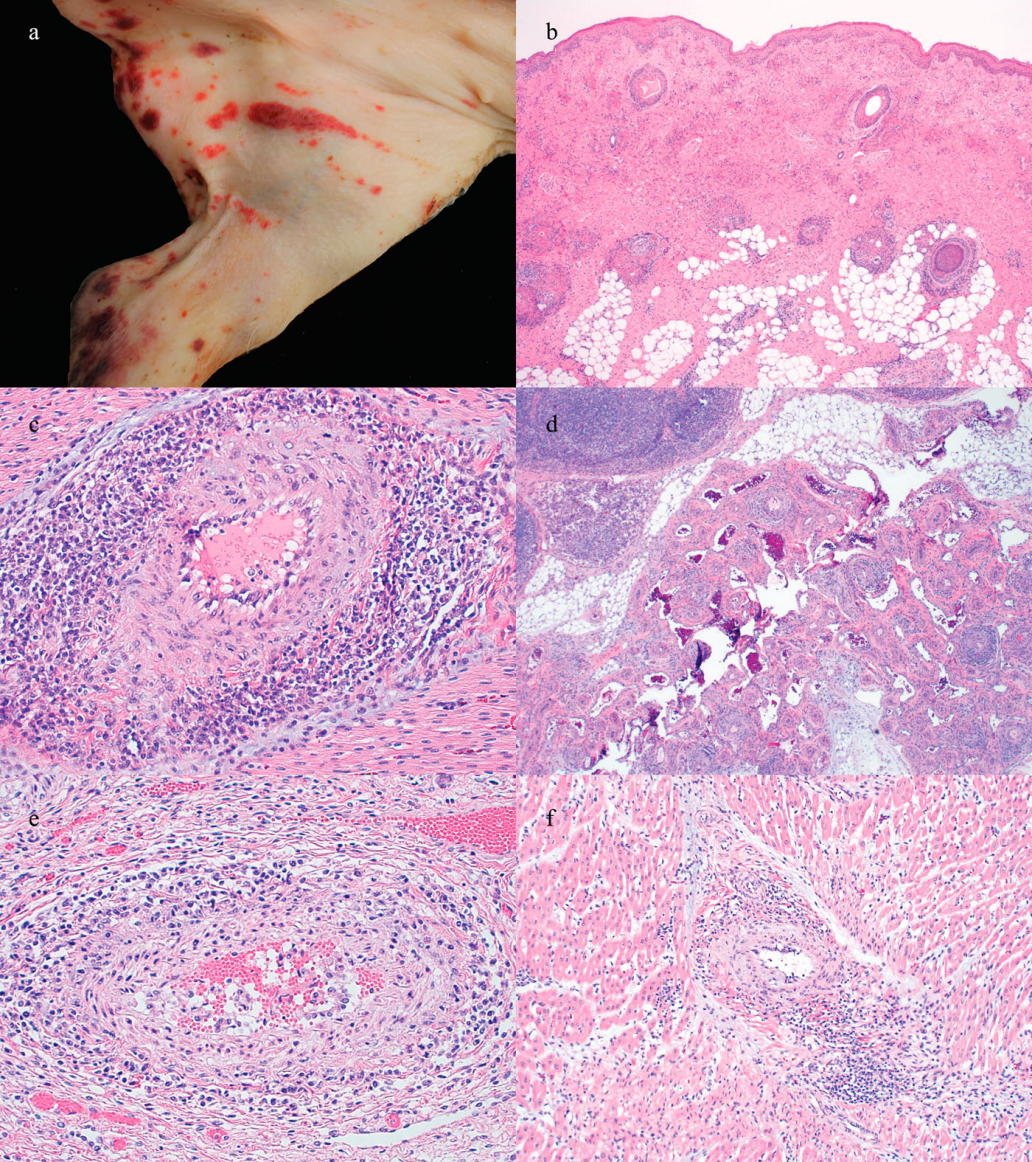
Gross and histologic lesions in weaned pig cases. (a) Palpable purpura and (b) epidermal necrosis, vasculitis and inflammation surrounding hair follicles in the 3-week-old pig from Case no. 2. (c) Lymphocytic periarteritis in the heart and (d) intestinal mesentery of the 25-day-old pig from Case no. 7. (e) Lymphocytic periarteritis in the kidney of the 5-week-old pig from Case no. 9. (f) Lymphocytic periarteritis and myocarditis in the 6-week-old pig from Case no. 8.

In two cases that consisted of 3-week-old pigs from two different sites supplied by the same sow farm, multifocal to coalescing, slightly raised, red to dark red, irregular, well-delineated macules and papules that occasionally had military central areas of depression (palpable purpura) were observed primarily in dependent areas (Figure 3(a)). Gross examination of other fresh and fixed tissues from other cases was commonly diagnostically unremarkable.

Histologic examination of the first two cases in which palpable purpura was observed, a leukocytoclastic vasculitis and fibrinoid necrosis with epidermal necrosis was present (Figure 3(b)). Mott cells and abundant macrophages were also present in lymph nodes. In the second case, a lymphohistiocytic and plasmacytic periarteritis and arteritis were present in the heart, kidney and mesenteric vessels. In the six other cases from distinct unrelated farms and three additional cases from the same flow that had previously observed palpable purpura, a predominately lymphocytic inflammatory infiltrate was commonly and concurrently present in the heart and kidney and less commonly concurrently in the mesentery (Figure 3(c–f)). In some cases, vasculitis was also present.

**Figure 4.**
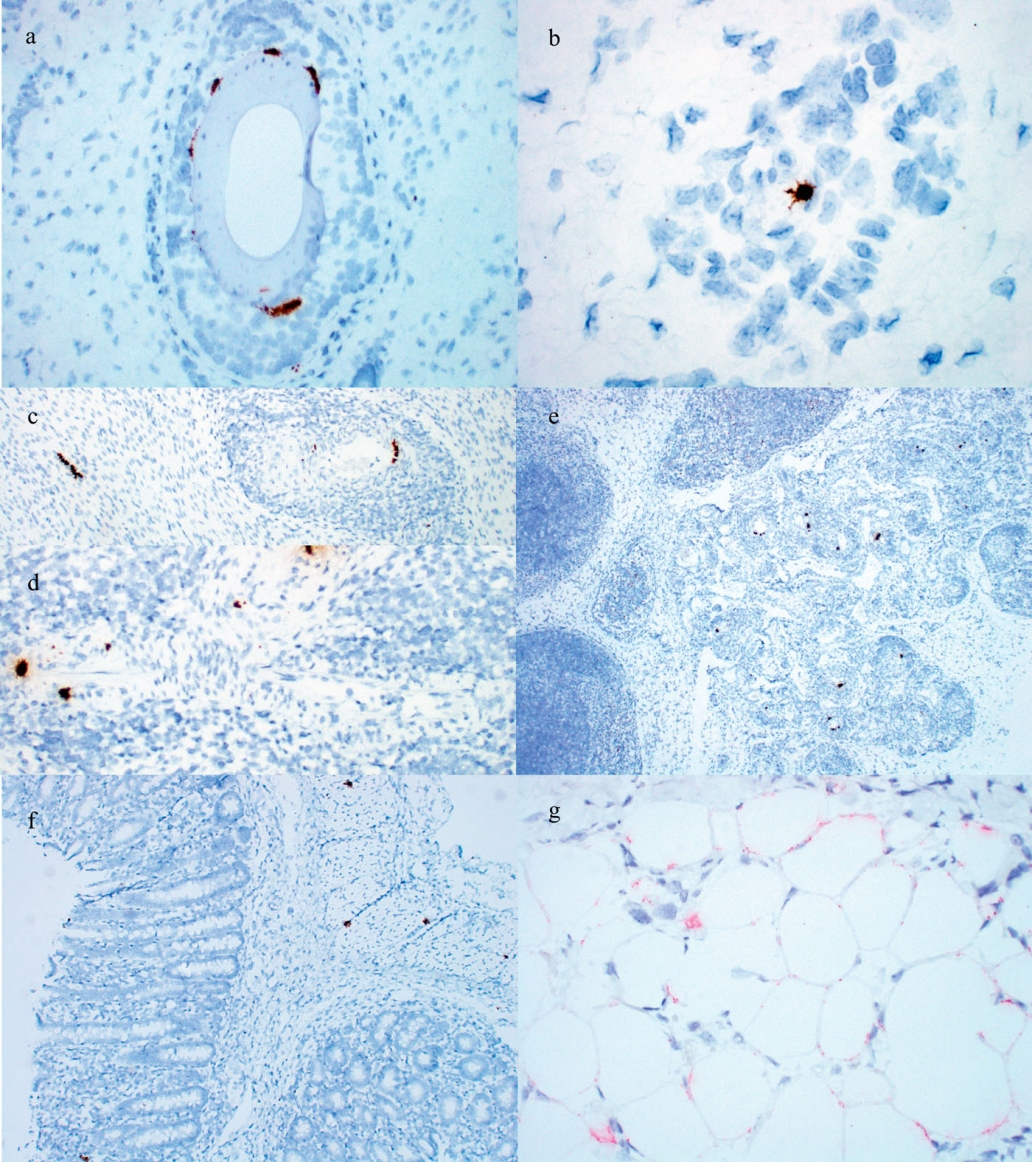
Detection of PCV3 by ISH in weaned pig cases. (a) PCV3 in hair follicle epithelium of the 3-week-old pig from Case no. 2. (b) PCV3 in an endothelial cell of a small arteriole in the dermis of the 3-week-old pig from Case no. 2. (c) PCV3 in cardiac myocytes and smooth muscle of an arteriole surrounded by inflammation in the 25-day-old pig from Case no. 7. (d) PCV3 in smooth muscle of an arteriole surrounded by inflammation in a 6-week-old pig from Case no. 8. (e) PCV3 in smooth muscle of multiple arterioles surrounded by inflammation and gut-associated lymphoid tissue of the 25-day-old pig from Case no. 7. PCV3 in the (f) smooth muscle and lamina propria of the large intestine and (g) adipocytes of the panniculus of the 3-week-old pig from Case no. 2.

PCV3 was detected by ISH in all cases. PCV3 was detected in the epidermis, follicular epithelium and arterioles in PDNS cases (Figure 4(a,b)). Replication of PCV3 was most commonly noted in the smooth muscle of arteries (Figure 4(c–e)) and less commonly in cardiac myocytes and gut-associated lymphoid tissue (Figure 4(c,e)). PCV3 was also observed in the smooth muscle of the large intestine and adipocytes (Figure 4(f,g)).

#### PCV3 detection in tissues by qPCR

PCV3 was detected in the lung of all affected pigs with an average Cq value of 24.3 (1.27 × 10^8 ^gc/mL) and a median Cq value of 23.6 (1.98 × 10^8 ^gc/mL). In four cases, multiple tissue types were assayed. PCV3 was commonly detected at a similar Cq value across multiple tissue types (Supplementary Table 3).

#### Viral pathogen detection in tissues by qPCR

PCV2 was not detected by qPCR in any case. PRRSV was not detected by RT-qPCR in most cases. PRRSV was detected by RT-qPCR in three cases (Cq 23.3, 31.4, and 31.1; PRRSV sequencing was not performed). PRRSV RT-qPCR was not performed in two cases.

#### PCV3 open reading frame 2 phylogenetic analysis

**Figure 5.**
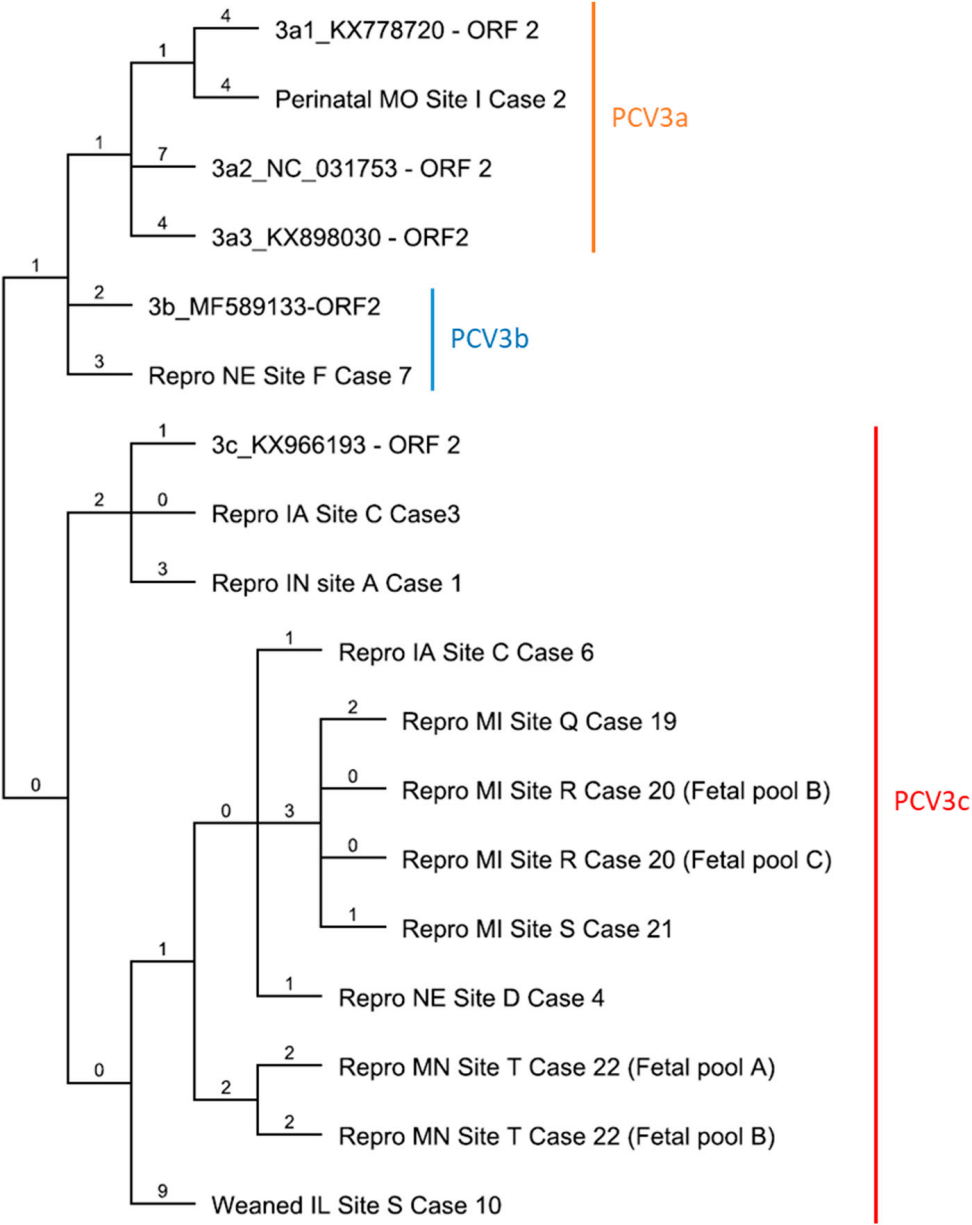
Phylogenetic tree of 11 clinical cases compared with 5 reference strains. Maximum parsimony (MP) tree based on the nucleotide sequences of the *cap* protein reconstructed using Geneious R9 software. The value along the branches represent substitutions per site.

**Figure 6.**
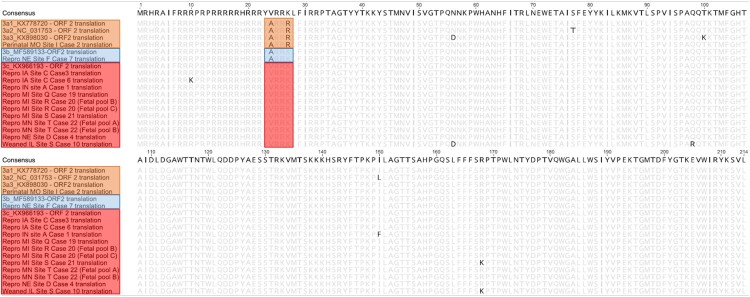
Full-length PCV3 *cap* protein from 11 clinical cases aligned by Geneious R9 software. Two amino acid mutations are used for PCV3 clade division (A24 V and R27 K). Strains in clade PCV3a, PCV3b and PCV3c are shown in the upper, middle and bottom box, respectively.

The ORF2 genomic sequences of 11 cases including 1 perinatal case, 1 weaned pig case and 9 reproductive failure cases were determined using either metagenomic or ORF2 sequencing. Phylogenetic analysis of the nucleotide sequence encoding the *cap* protein was performed comparing four reference strains representing all PCV3 clades and three PCV3a subclades. Based on the ORF2 nucleotide sequence of three PCV3a reference strains, one perinatal infection case belonged to the PCV3a clade. The percent identity of the ORF2 genome of this individual case compared with reference sequences of PCV3a1, PCV3a2 and PCV3a3 was 98.76%, 98.14%, and 98.60%, respectively. Only one reproductive case was represented within the clade PCV3b with a 99.2% nucleotide identity compared with a reference strain. Eight reproductive failure cases and one weaned pig case were represented within the PCV3c clade. The percentage of nucleotide identity amongst reproductive failure cases within the PCV3b clade varied from 98.76% to 99.84%. The only weaned pig case evaluated had the lowest homology with the PCV3c reference strain (98.14%) (Figure 5). In addition, genetic differences resulted in amino acid mutations (A24 V and R27 K) represented with one perinatal case (A24: R27), one weaned pig case (A24:K27), and nine reproductive failure cases (V24:K27; Figure 6). These results further validate the genotyping results that were obtained based on the phylogenetic structure.

## Discussion

PCV2 was initially but sporadically identified in cases of PCV2-SD in Canada in the 1990s [5, 8]; however, epidemics of this severe systemic disease due to PCV2 were not seen in the global swine population until the ensuing decade [9–14]. Although there are effective commercially available vaccines, PCV2 remains an endemic infection that can cause several disease syndromes of economic significance in swine [6,7]. The clinical and histopathologic manifestations of PCV2 infection include mummies and stillbirths, myocarditis in fetuses, interstitial pneumonia with peribronchiolitis, granulomatous lymphadenitis with lymphoid depletion, PDNS, and enteritis; however, clinical signs and lesions occur in only a portion of infected animals [6,7]. Similar to PCV2, PCV3 can be found in apparently healthy and diseased pigs [43,44].

In this report, PCV3 was identified by qPCR in mummified and stillborn fetuses in 22 cases that included 36 fetuses or fetal groups from multiple production systems located in different states and 20 different sites. PCV2, which has previously been associated with these clinical presentations, was not identified. High levels of PCV3 nucleic acid were detected in mummified and stillborn fetuses by qPCR, with Cq values suggesting high viral titers in the billions to trillions of genome copies per mL of tissue homogenate. Metagenomic sequencing was performed on fetal thoracic tissue in eight of these cases that included sixteen fetal pools, PCV3 was the only virus identified in fourteen fetal pools. Histopathologic examination of a majority of fetuses was unremarkable, which is not unusual with viral causes of reproductive failure or *in utero* death. However, lymphocytic myocarditis was noted in two cases, which is similar to what has been observed in PCV2-associated reproductive failure [6,7]. Additionally, ISH provides evidence for replication of PCV3 within sections of fetal heart, kidney, placenta and the smooth muscle of arteries, with the most abundant nucleic acid detection present in a fetus with lymphocytic myocarditis. In fetuses, microscopic lesions associated with PCV2, when present, are most prominent in the heart making this the organ of choice for diagnostic work-up of PCV2-induced fetal deaths [6]. PCV3 appears to be similar to PCV2 in this regard. The exact timing of cardiac lesions in fetuses is difficult to determine as time of exposure of the fetus depends on dam viremia and spread to the fetus either through the placenta or from adjacent fetuses. The crown to rump length of mummified fetuses ranged from 7 to 17 cm suggesting *in utero* fetal death occurred sometime between 45 and 70 days of gestation.

To compare frequency of detection and to estimate relative frequency of common causes of reproductive failure as determined by fetal thoracic tissue testing, Iowa State University Veterinary Diagnostic Laboratory (ISU-VDL) data were reviewed. During a 10 month period (1 January 2018 to 10 January 2018), PRRSV, PCV2, PCV3 or PPV were detected by PCR in fetal tissues at a Cq value of less than or equal to 30 in 63 cases. PRRSV was detected in 38 of the 347 cases tested (11% of tested cases positive). PCV3 was detected in 18 of 138 cases tested (13% positive). PPV and PCV2 were detected in 7 of 140 and 10 of 200 cases tested (5% positive), respectively. Concurrent detection of these viral agents in a subset of cases not included above did occur (PRRSV/PCV2: *n* = 2; PCV2/PCV3: *n* = 2; PPV/PRRSV: *n* = 1; PPV/PCV2: *n* = 1; and PPV/PCV3: *n* = 1). Although limited, this information suggests that PCV3 has an impact on the fetal wastage in the United States swine herd.

PCV3 was also detected in high quantities in the brain of neonatal pigs in three cases. Cq values of 7.5–13.3 in the cerebrum indicate high viral titers of billions to trillions of genome copies per mL of tissue homogenate. Lymphocytic perivascular cuffs and gliosis were noted in the two cases in which cerebrum was evaluated histologically. APPV, experimentally demonstrated to be a cause of congenital tremors [42,45], was also identified by RT-qPCR in the two cases in which tremors were reported; however, piglets with APPV-associated congenital tremors do not have inflammation present in their cerebrum due to the pathophysiology of the virus–host interaction [2]. The possibility of a synergistic relationship between PCV3 and APPV requires further evaluation. Lymphocytic myocarditis and perivasculitis were also observed in two cases with PCV3 replication noted in the cardiac myocytes and smooth muscle of arteries. These findings are very similar to a report of an 8-day-old pig with myocarditis due to PCV2 [46]. As with PCV2, PCV3 appears to have a proclivity for the cardiovascular system.

PCV3 was also detected by qPCR consistently in the lung of growing pigs with periarteritis and arteritis in multiple tissues similar to what has been previously reported [23]. PCV3 was also detected by ISH in the smooth muscle of arteries and arterioles of all cases. Severe diffuse segmental to circumferential lymphohistiocytic and plasmacytic periarteritis and endarteritis in several organs has also been reported in a pig naturally infected with PCV2 [47]. In that report, the affected arterioles were observed in the heart, meninges, lungs, medulla and pelvis of the kidneys and intestinal mesentery and PCV2 was detected by immunohistochemistry in smooth muscle-like cells of arteries. The presence of PCV3 was not evaluated in that report, and severe polyarteritis in a single pig with PCV2 as was reported is not a frequent observation in pigs submitted to diagnostic laboratories with PCV2-associated disease [47].

PCV3 was also detected in pigs with gross and histologic lesions consistent with PDNS. Interestingly, Mott cells were present in the lymph nodes of these pigs. Mott cells may be present in neoplastic conditions; however, they can be commonly found in situations of chronic and/or intense antigen stimulation [47]. The cause or causes of intense antigen stimulation in these pigs were not identified but perhaps represent a risk factor in the development of PDNS.

In all cases assayed, PCV3 replication was noted in the smooth muscle of arteries. Based on this evidence, virus isolation of PCV3 in a smooth muscle cell culture may be rewarding and perhaps additional research should focus on the interaction of PCV3 and arteries/arterioles. Interestingly, PCV3 was also noted in adipocytes. The implications of this finding are currently unknown and require further investigation.

It has been previously proposed that mutations in the amino acids 24 and 27 of the *cap* protein could be potential molecular markers to identify different PCV3 clades [37]. Phylogenetic analysis of the ORF2 demonstrated that clinical cases evaluated in this study are represented in all three PCV3 clades. A majority of the reproductive cases evaluated in this study belonged to the PCV3c clade. The only PCV3c US reference strain (PCV3/USA/SD2016/2016(KX966193)) was originally described in cases of multisystemic inflammation [23]. A single reproductive failure case was characterized as PCV3b. Although in the United States there are a few strains reported to belong to the PCV3b clade, this clade seems to be widely distributed in China. PCV3a was associated with disease in a single perinatal infection case [37,48]. Based on the ORF2 analysis, all three PCV3 clades are circulating in the United States swine herd and strongly associated with disease.

Similar to PCV2, the detection of PCV3 in retrospective samples indicates that PCV3 has likely been circulating in swine populations decades prior to the initial reports [36]. However, the seroprevalence and association with disease over time remains unknown. As with any endemic agent, expression of disease will be highly dependent on the seroprevalence of the herd and coinfections. It is expected that as testing increases, PCV3 will be identified in more countries and more retrospective sample sets.

Since the initial descriptions of PCV3-associated disease, there has been a sparsity of detection of PCV3 in lesions with many of the publications focused on detection of PCV3 by PCR. Detection of an endemic agent alone does not suffice in the diagnosis of disease. Using histopathology and ISH, this report documents PCV3 as a putative cause of reproductive failure as well as multisystemic inflammation in perinatal and grow-finish pigs derived from multiple US swine farms and production systems, with some features similar to those reported with PCV2-associated PCVAD in the absence of PCV2. Retrospective studies suggest that PCV2 infection resulted in sporadically systemic disease as earlier as 1985 before devastating epidemics were observed in the late 1990s and was present in some populations at least since 1962 [11,49]. PCV3-associated disease may also follow a similar course. Based on the evidence presented, further studies investigating the pathophysiology, epidemiology, immunopathogenesis, and economic impact of PCV3 are warranted [50–53].

## Supplementary Material

Supplemental Material

## References

[1] Breitbart M, Delwart E, Rosario K, et al. ICTV Virus Taxonomy: Circoviridae. J Gen Virol 2017;98:1997–1998.

[2] Segalés J, Allan GM, Domingo M, et al. Diseases of swine. 10th edn West Sussex: Wiley-Blackwell; 2012 Chapter 26.

[3] Cheung AK. Comparative analysis of the transcriptional patterns of pathogenic and nonpathogenic porcine circoviruses. Virology. 2003;310:41–49. doi: 10.1016/S0042-6822(03)00096-5 12788629

[4] Clark EG. Pathology of postweaning multisystemic wasting syndrome of pigs. Proc West Can Assoc Swine Prod. 1996: 22–25.

[5] Harding JCS, Clark EG, Strokappe JH, et al. Postweaning multisystemic wasting syndrome: epidemiology and clinical presentation. Swine Health Prod. 1998;6:249–254.

[6] Opriessnig T, Langohr I. Current state of knowledge on porcine circovirus type 2-associated lesions. Vet Pathol. 2013;50:23–38. doi: 10.1177/0300985812450726 22692624

[7] Segalés J. Porcine circovirus type 2 (PCV2) infections: clinical signs, pathology and laboratory diagnosis. Virus Res. 2012;164:10–19. doi: 10.1016/j.virusres.2011.10.007 22056845

[8] Ellis J. Porcine circovirus: a historical perspective. Vet Pathol. 2014;51:315–327. doi: 10.1177/0300985814521245 24569612

[9] Allan GM, McNeilly F, Kennedy S, et al. Isolation of porcine circovirus-like viruses from pigs with a wasting disease in the USA and Europe. J Vet Diagn Invest. 1998;10:3–10. doi: 10.1177/104063879801000102 9526853

[10] Allan G, McNeilly F, Walker IW, et al. Novel porcine circoviruses from pigs with wasting disease syndromes. Vet Rec. 1998;142:467–468. doi: 10.1136/vr.142.1.8 9602519

[11] Sato K, Shibahara T, Ishikawa Y, et al. Evidence of porcine circovirus infection in pigs with wasting disease syndrome from 1985 to 1999 in Hokkaido, Japan. Journal of Veterinary Medical Science. 2000;62:627–633. doi: 10.1292/jvms.62.627 10907690

[12] Ellis J, Hassard L, Clark E, et al. Isolation of circovirus from lesions of pigs with postweaning multisystemic wasting syndrome. Can Vet J. 1998;39:44–51. 9442952 PMC1539838

[13] Segalés J, Sitjar M, Domingo M, et al. First report of post-weaning multisystemic wasting syndrome in pigs in Spain. Vet Rec. 1997;141:600–601. 9429277

[14] Choi C, Chae C, Clark EG. Porcine postweaning multisystemic wasting syndrome in Korean pig: detection of porcine circovirus 2 infection by immunohistochemistry and polymerase chain reaction. J Vet Diagn Invest. 2000;12:151–153. doi: 10.1177/104063870001200209 10730945

[15] Allan GM, McNeilly F, Ellis J, et al. PMWS: experimental model and co-infections. Vet Microbiol. 2004;98:165–168. doi: 10.1016/j.vetmic.2003.10.009 14741129

[16] Drolet R, Thibault S, D'Allaire S, et al. Porcine dermatitis and nephropathy syndrome (PDNS): an overview of the disease. Swine Health Prod. 1999;7:283–285.

[17] West KH, Bystrom JM., Wojnarowicz Chris, et al. Myocarditis and abortion associated with intrauterine infection of sows with porcine circovirus 2. J Vet Diagn Invest. 1999;11:530–532. doi: 10.1177/104063879901100608 12968736

[18] Brunborg IM, Jonassen CM., Moldal T, et al. Association of myocarditis with high viral load of porcine circovirus type 2 in several tissues in cases of fetal death and high mortality in piglets. A case study. J Vet Diagn Invest. 2007;19:368–375. doi: 10.1177/104063870701900405 17609345

[19] Pittman JS. Reproductive failure associated with porcine circovirus type 2 in gilts. J Swine Health Prod. 2008;16:144–148.

[20] Segalés J, Allan GM, Domingo M. Porcine circovirus diseases. Anim Health Res Rev. 2005;6:119–142. doi: 10.1079/AHR2005106 16583778

[21] Hansen MS, Hjulsager CK., Bille-Hansen V, et al. Selection of method is crucial for the diagnosis of porcine circovirus type 2 associated reproductive failures. Vet Microbiol. 2010;144:203–209. doi: 10.1016/j.vetmic.2009.12.038 20097019

[22] Palinski R, Piñeyro P, Shang P, et al. A novel porcine circovirus distantly related to known circoviruses is associated with porcine dermatitis and nephropathy syndrome and reproductive failure. J Virol. 2017;91:e01879–16. doi: 10.1128/JVI.01879-16 27795441 PMC5165205

[23] Phan TG, Giannitti F, Rossow S, et al. Detection of a novel circovirus PCV3 in pigs with cardiac and multi-systemic inflammation. Virol J. 2016;13:184. doi: 10.1186/s12985-016-0642-z 27835942 PMC5105309

[24] Fux R, Söckler C, Link EK, et al. Full genome characterization of porcine circovirus type 3 isolates reveals the existence of two distinct groups of virus strains. Virol J. 2018;15:25. doi: 10.1186/s12985-018-0929-3 29378597 PMC5789634

[25] Hayashi S, Ohshima Y, Furuya Y, et al. First detection of porcine circovirus type 3 in Japan. J Vet Med Sci. 2018;80:1468–1472. doi: 10.1292/jvms.18-0079 30078831 PMC6160883

[26] Kim SH, Park JY, Jung Jung, et al. Detection and genetic characterization of porcine circovirus 3 from aborted fetuses and pigs with respiratory disease in Korea. J Vet Sci. 2018;19:21–724. doi: 10.4142/jvs.2018.19.1.21 30041289 PMC6167340

[27] Yuzhakov AG, Raev SA., Alekseev KP, et al. First detection and full genome sequence of porcine circovirus type 3 in Russia. Virus Genes. 2018;54:608–611. doi: 10.1007/s11262-018-1582-z 29948781

[28] Zhao D, Wang X, Gao Q, et al. 2018 Retrospective survey and phylogenetic analysis of porcine circovirus type 3 in Jiangsu province, China, 2008 to 2017. Arch Virol. 2018;163:2531–2538. 10.1007/s00705-018-3870-2 29802547

[29] Kedkovid R, Woonwong Y, Arunorat J, et al. Porcine circovirus type 3 (PCV3) infection in grower pigs from a Thai farm suffering from porcine respiratory disease complex (PRDC). Veterinary Microbiology. 2018;215:71–76. doi: 10.1016/j.vetmic.2018.01.004 29426409

[30] Faccini S, Barbieri I., Gilioli A., et al. Detection and genetic characterization of porcine circovirus type 3 in Italy. Transbound Emerg Dis. 2017;64:1661–1166. doi: 10.1111/tbed.12714 28921870

[31] Franzo G, Legnardi M, Hjulsager CK, et al. Full-genome sequencing of porcine circovirus 3 field strains from Denmark, Italy and Spain demonstrates a high within-Europe genetic heterogeneity. Transbound Emerg Dis. 2018;65:602–606. 10.1111/tbed.12836 29453822

[32] Kwon T, Yoo SJ, Park CK, et al. Prevalence of novel porcine circovirus 3 in Korean pig populations. Vet Microbiol. 2017;207:178–180. doi: 10.1016/j.vetmic.2017.06.013 28757021

[33] Stadejek T, Wozniak A, Milek D, et al. First detection of porcine circovirus type 3 on commercial pig farms in Poland. Transbound Emerg Dis. 2017;64:1350–1353. doi: 10.1111/tbed.12672 28649803

[34] Tochetto C, Lima DA, Varela APM, et al. Full-genome sequence of porcine circovirus type 3 recovered from serum of sows with stillbirths in Brazil. Transbound Emerg Dis. 2018;65:5–9. doi: 10.1111/tbed.12735 29027372

[35] Ye X, Berg M, Fossum C, et al. Detection and genetic characterisation of porcine circovirus 3 from pigs in Sweden. Virol Sin. 2018;54:466–469. 10.1007/s11262-018-1553-4 PMC5951868 29564688

[36] Ku X, Chen F, Li P, et al. Identification and genetic characterization of porcine circovirus type 3 in China. Transbound Emerg Dis. 2017;64:703–708. doi: 10.1111/tbed.12638 28317326 PMC7169768

[37] Fu X, Fang B, Ma J, et al. Insights into the epidemic characteristics and evolutionary history of the novel porcine circovirus type 3 in southern China. Transbound Emerg Dis. 2018;65:296–303. doi: 10.1111/tbed.12752 29178283

[38] Zhai SL, Zhou X, Zhang H, et al. Comparative epidemiology of porcine circovirus type 3 in pigs with different clinical presentations. Virol J. 2017;14:222. doi: 10.1186/s12985-017-0892-4 29132394 PMC5683367

[39] Shen H, Liu X, Zhang P, et al. Genome characterization of a porcine circovirus type 3 in South China. Transbound Emerg Dis. 2018;6:264–266. doi: 10.1111/tbed.12639 28296271

[40] Opriessnig T, Yu S, Gallup JM, et al. Effect of vaccination with selective bacterins on conventional pigs infected with type 2 porcine circovirus. Vet Pathol. 2003;40:521–529. doi: 10.1354/vp.40-5-521 12949409

[41] Chen HY, Li X-K, Cui B-A, et al. A TaqMan-based real-time polymerase chain reaction for the detection of porcine parvovirus. J Virol Methods. 2009;156:84–88. doi: 10.1016/j.jviromet.2008.10.029 19041671

[42] Arruda BL, Arruda PH, Magstadt DR, et al. Identification of a divergent lineage porcine pestivirus in nursing piglets with congenital tremors and reproduction of disease following experimental inoculation. PLoS One. 2016;11:e0150104. doi: 10.1371/journal.pone.0150104 26909691 PMC4766193

[43] Kedkovid R, Woonwong Y, Arunorat J, et al. Porcine circovirus type 3 (PCV3) shedding in sow colostrum. Vet Microbiol. 2018;220:12–17. doi: 10.1016/j.vetmic.2018.04.032 29885795

[44] Zheng S, Wu X, Zhang L, et al. The occurrence of porcine circovirus 3 without clinical infection signs in Shandong Province. Transbound Emerg Dis. 2017;64:1337–1341. doi: 10.1111/tbed.12667 28653486 PMC7169790

[45] de Groof A, Deijs M, Guelen L, et al. Atypical porcine pestivirus: a possible cause of congenital tremor type A-II in newborn piglets. Viruses. 2016;8:271. doi: 10.3390/v8100271 PMC5086607 27782037

[46] Mikami O, Nakajima H, Kawashima K, et al. Nonsuppurative myocarditis caused by porcine circovirus type 2 in a weak-born piglet. J Vet Med Sci. 2005;67:735–738. doi: 10.1292/jvms.67.735 16082126

[47] Opriessnig T, Janke BH, Halbur PG. Cardiovascular lesions in pigs naturally or experimentally infected with porcine circovirus type 2. J Comp Pathol. 2006;134:105–110. doi: 10.1016/j.jcpa.2005.06.007 16325842

[48] Li G, He W, Zhu H, et al. Origin, genetic diversity, and evolutionary dynamics of novel porcine circovirus 3. Adv Sci. 2018;5:1800275. doi: 10.1002/advs.201800275 PMC6145280 30250786

[49] Jacobsen B, Krueger L, Seeliger F, et al. Retrospective study of the occurrence of porcine circovirus 2 infection and associated entities in Northern Germany. Vet Microbiol. 2009;138:27–33. doi: 10.1016/j.vetmic.2009.02.005 19268497

[50] Cazzini P, Watson VE, Brown HM. The many faces of Mott cells. Vet Clin Pathol. 2013;42:125–126. doi: 10.1111/vcp.12043 23731000

[51] Hause BM, Collin EA, Anderson J, et al. Bovine rhinitis viruses are common in U. S. cattle with bovine respiratory disease. PLoS One. 2015;10:e0121998. doi: 10.1371/journal.pone.0121998 25789939 PMC4366061

[52] Neill JD, Bayles DO, Ridpath JF. Simultaneous rapid sequencing of multiple RNA virus genomes. Journal of Virological Methods. 2014;201:68–72. doi: 10.1016/j.jviromet.2014.02.016 24589514 PMC7119728

[53] Allander T, Tammi MT, Eriksson M, et al. Cloning of a human parvovirus by molecular screening of respiratory tract samples. Proc Natl Acad Sci USA. 2005;102:12891–12896. doi: 10.1073/pnas.0504666102 16118271 PMC1200281

