# Supplementary material for: PCV3-associated disease in the United States swine herd
Source: Emerg Microbes Infect. 2019 May 16;8(1):684–98. doi: 10.1080/22221751.2019.1613176 (PMC6534263; doi:10.1080/22221751.2019.1613176)
Supplement: Supplemental Material [file TEMI_A_1613176_SM3319.zip › Supplementary Table 1.docx]

Supplementary Table 1. PCV3 qPCR Cq values by tissue type of reproductive failure cases.

| **Case No.** | **Group ID** | **PCV3 Cq**^1^ | | | |
| --- | --- | --- | --- | --- | --- |
|  |  | **Fetal thoracic tissue**^2^ | **Fetal thoracic fluid** | **Kidney** | **Liver** |
| 1 | NA^3^ | 20.6 | ND^4^ | 21.6 | ND |
| 3 | NA | 9.4 | ND | 12.5 | ND |
| 6 | A | 14.8 | 12.7 | 17.1 | 13.4 |
| 6 | B | 30.7 | 25.6 | 34.4 | U^5^ |
| 6 | C | 10.8 | 9.45 | 15.2 | 12.1 |
| 8 | NA | 18 | ND | 20.7 | ND |

^1^Cq: Cycle quantification value. ^2^Fetal thoracic tissue: Pooled lung and heart. ^3^NA^:^ Not applicable.

^4^ND: Not done. ^5^U: Not detected after 40 cycles.
