# Supplementary material for: PCV3-associated disease in the United States swine herd
Source: Emerg Microbes Infect. 2019 May 16;8(1):684–98. doi: 10.1080/22221751.2019.1613176 (PMC6534263; doi:10.1080/22221751.2019.1613176)
Supplement: Supplemental Material [file TEMI_A_1613176_SM3319.zip › Supplementary Table 2.docx]

Supplementary Table 2. Summary of metagenomics results from reproductive failure cases.

| **Case No.** | **Fetal Pool ID** | **No. of Sequences** | **PCV3 (% Whole Genome)** | **PCV3 Identity^1^** | **TTV^2^ (% Genome)** | **TTV Identity** | **Porcine CMV^3^ (% Genome)** | **Porcine CMV Identity** |
| --- | --- | --- | --- | --- | --- | --- | --- | --- |
| 1 | A | 468 | 100.0% | MF162299 | 0% | NA | 0% | NA |
| 3 | A | 652,457 | 100.0% | MF162299 | 0% | NA | 0% | NA |
| 6 | A | 348,835 | 100.0% | MG564174 | 0% | NA | 0% | NA |
| 7 | A | 1,370 | 100.0% | MF162299 | 0% | NA | 0% | NA |
| 19 | A | 118,911 | 31.6% | MG868941 | 0% | NA | 0% | NA |
|  | B | 148,934 | 100% | MG696866 | 0% | NA | 0% | NA |
|  | C | 63,320 | 24.6% | MG868943 | 0% | NA | 0% | NA |
| 20 | A | 80,696 | 96.1% | MG696866 | 0% | NA | 0% | NA |
|  | B | 45,258 | 100% | MG696866 | 0% | NA | 0% | NA |
|  | C | 187,516 | 100% | MG696866 | 0% | NA | 0% | NA |
|  | D | 69,225 | 66.9% | MG868946 | 0% | NA | 99.50% | KF017583^4^ |
| 21 | A | 122,496 | 16.7% | MG868943 | 0% | NA | 0% | NA |
|  | B | 80,473 | 100% | MG696866 | 92.6% | KT968712^5^ | 0% | NA |
| 22 | A | 213,727 | 100% | MH192341 | 0% | NA | 0% | NA |
|  | B | 241,648 | 100% | MH192341 | 0% | NA | 0% | NA |
|  | C | 223,398 | 18.6% | MG868943 | 0% | NA | 0% | NA |

^1^All PCV3 sequences exhibited 99-100% identity to GenBank Reference. ^2^TTV: Torque teno virus. ^3^CMV: Cytomegalovirus. ^4^TTV exhibited 96% identity to GenBank Reference. ^5^Porcine CMV exhibited greater than 99% identity to GenBank Reference.
