# Supplementary material for: PCV3-associated disease in the United States swine herd
Source: Emerg Microbes Infect. 2019 May 16;8(1):684–98. doi: 10.1080/22221751.2019.1613176 (PMC6534263; doi:10.1080/22221751.2019.1613176)
Supplement: Supplemental Material [file TEMI_A_1613176_SM3319.zip › Supplementary Table 3.docx]

| **Case No.** | **Site ID** | **Age**  **(wk)** | **PCV3 Cq** |
| --- | --- | --- | --- |
| 2 | L | 3 | 26.5-Lung 23.0-Lymph node 23.5-Heart 22.1-Kidney 22.2-Liver |
| 4 | M | 8 | 25.8-Lung 28.7-Kidney 24.8-Liver 35.9-Heart |
| 5 | M | 9 | 27.0-Lung 25.9-Kidney |
| 6 | N | 10 | 28.0-Lung 25.8-Heart U-Liver |

Supplementary Table 3. PCV3 qPCR Cq values by tissue type of weaned pig cases.
